# Mathematical Model of the Pulse Generation of Decontaminating Aerosols

**DOI:** 10.3390/ma15228215

**Published:** 2022-11-18

**Authors:** Olga Kudryashova, Sergei Sokolov, Ilya Zhukov, Alexander Vorozhtsov

**Affiliations:** 1 Laboratory for High Energy and Special Materials, National Research Tomsk State University, Lenin Avenue, 36, 634050 Tomsk, Russia; 2 Institute for Problems of Chemical and Energy Technologies Siberian Branch of the Russian Academy of Sciences, St. Socialist, 1, 659322 Biysk, Russia; 3 Laboratory of Metallurgy Nanotechnologies, National Research Tomsk State University, Lenin Avenue, 36, 634050 Tomsk, Russia

**Keywords:** pulse generation, aerosol, decontamination, high-energy materials, mathematical model

## Abstract

A mathematical model of the pulse generation of decontaminating aerosols utilizing the energy of high-energy materials (HEM) is proposed with account for the physical and chemical properties of the atomized substance, HEM characteristics, and gas generator parameters. Such a model is needed to counter the environmental hazards, process emissions, and terrorist attacks with hazardous and dangerous aerosols. Another aspect of the problem is the danger of biological aerosols carrying viral or microbial particles that are spread naturally or induced using biological weapons. In many cases, the mission is not only to neutralize aerosol particles in indoor air and on surfaces but also to do it quickly. In this regard, an attractive option is the pulse method for generating special aerosols aimed at quickly, within a few seconds, creating a cloud of particles that will interact with hazardous aerosol particles and decontaminate them. HEM energy is proposed to be used for the pulse generation of such aerosols. It is important not only to atomize the decontaminating aerosol quickly and evenly in space but also to preserve the useful physical and chemical properties of the particles. To test the regimes and methods of pulse generation, an adequate mathematical model of the process is required, which is proposed in this manuscript.

## 1. Introduction

Decontamination, in a broad sense, is referred to as the removal of harmful and hazardous substances from the atmosphere and surfaces, i.e., detoxification, disinfection, and neutralization. These may be implemented by chemical agents, such as chlorine and other chemical disinfectants, or by physical methods, for example, ultraviolet irradiation [1,2], air filtration, ozonation, plasma and electrical discharges [3,4], etc. [5].

Air purification by filtration is probably the most studied method for removing the hazardous substances or microorganisms from indoor air. For example, an HEPA (High-Efficiency Particulate Air) filter is capable of capturing particles with a size of 2 µm or less. The filter is composed of fiberglass with diameters of 0.1–10 µm; the air space between fibers is greater than the size of the particles to be captured. During the air filtration, the particles are captured by the fibers and held on their surfaces due to electrostatic effects. Nowadays, new filtration methods are being developed, such as electrodynamic-based ones, which may be calibrated to selectively remove particles that are usually poorly removed by other methods, for example, in the range from 0.1 to 1 μm [6].

Traditional methods of decontamination are often limited by their inability to effectively inactivate a significant percentage of chemically hazardous particles, bacteria, and viruses, and have other disadvantages. For instance, chemical disinfectants provide harmful by-products after surface treatment [7]. Ultraviolet disinfection requires a long processing time; meanwhile, people and animals, which may be injured by radiation, should be removed. Such a treatment is ineffective for the decontamination of “blind” zones shielded by objects. Moreover, ultraviolet irradiation may lead to serious infections due to the photoreactivation of pathogenic bacteria [8].

Nowadays, electrostatic atomization is considered as a traditional method of decontamination [9]. This method has shown excellent results in decontaminating large open areas, such as airports, waiting areas, classrooms, gyms, and portable equipment [10]. Another advantage of this method is its ability to remove hazardous substances from hard-to-reach surfaces. However, it requires a long processing time and accurate equipment settings [11].

The ultrasonic atomization of disinfectants is also a method that is widely used and is constantly being developed. The successful application of a composition containing bacteriophages, bacterial spores, and bacterial cells in the disinfection of textiles and footwear is described in [12]. Disinfection is performed in a closet using an ultrasonic nebulizer. This device atomizes disinfectants using a cavitation aerosol-generating procedure. The method is rather effective but time-consuming.

To disinfect large areas, traditional automated atomizers, the so-called no-touch automated disinfection systems, are also applied [13]. The widely used systems of this type are aerosolized hydrogen peroxide (aHP) atomizers that deliver a pressure-generated aerosol [14]. The systems most encountered in healthcare use a solution containing 5–6% hydrogen peroxide and <50 parts per million of silver [15]. Aerosolized droplets of submicron and micron sizes are introduced into an enclosure through a unidirectional nozzle [16]. The dose typically recommended for hospital rooms is 6 ml/m^3^. After exposure, the aerosol usually decomposes naturally.

The available innovative disinfection methods are described in a review [11]. These methods imply the preliminary application of antimicrobial spray nanocoatings [17] or the formation of self-disinfection surfaces [18]. The latter is based on photocatalysis together with the physical removal of particles by membrane filters.

The work [19] presents a method based on electrostatic forces using silver nanoparticles and polyethyleneimine (size of 6.2 ± 2.6 nm) to generate antiviral coatings on filter materials with an inactivation yields against SARS-CoV-2 of greater than 99.9%.

The photosensitivity effect is used for innovative therapeutic applications [20]. Light-sensitive iron oxide nanoparticles embedded in biocompatible electrospun nanofibers induce membrane permeabilization by photothermal effects. The photothermal nanofibers have been successfully used to deliver effector molecules to cells.

The development of new materials for wound dressing without antibiotics is important [21,22,23]. For healing and the disinfection of wounds, “nanoparticle dressings” are included into scaffolds. Silver nanoparticles (Ag-NPs) are a promising material for wound healing because of their excellent antimicrobial properties.

Most of the traditional or innovative decontamination methods are specific (aimed at removing either microorganisms or chemicals from air, water, or surfaces) and/or have some limitations. Many of them are obliged to isolate people and animals from the area during processing and take much time, while others are ineffective at the removing hazardous substances and may leave destructive byproducts.

In certain circumstances, the rapid removal of hazardous agents from the atmosphere and indoor surfaces is needed. In such cases, the methods of pulse atomization of special decontaminating aerosols, followed by the disposal of the spent aerosol particles, may be useful. Such situations arise after an accidental release of gases/aerosols, during the terrorist attacks, when dangerous infections are detected indoors, etc. nanocoatings. 

The proposed pulse method of atomization of decontaminating aerosols has the following advantages:

- Speed of aerosol creation;
- Non-specific—can be used to remove both microorganisms and chemicals from surfaces and from the air.

The main disadvantage of the pulse atomization method is the need to develop a sprayer design that provides a guaranteed disinfecting effect. The effect depends on the size of the sprayed particles—the smaller they are, the larger the surface of interaction with harmful agents, the better. Secondly, it is necessary that the disinfectant does not lose its properties as a result of an aggressive pulsed spraying method.

Three phases are possible for atomized aerosols: solid (powder), liquid, and heterophase (liquid/powder mixture). The decontamination procedure may differ in terms of the object to be neutralized and the area to be treated (enclosed or semi-enclosed room, open space). Aerosol particles or droplets may adsorb toxic agents or gases, react with them, have catalytic activity to decompose hazardous substances, destroy/deactivate microorganisms, etc.

Therefore, when developing methods of decontamination with aerosol clouds, it is necessary to account for the phase composition of the aerosol and the transformations of the particles during the atomization process. 

The first stage in the development of such methods of atomization is mathematical and physical modeling. This step will allow us to determine the desired parameters of the atomizer to achieve the best effect. As mentioned above, it is necessary to achieve the minimum particle size of the sprayed substance.

This work aims to propose a mathematical model of the pulse generation of a decontaminating aerosol with account for the phase composition and physical and chemical properties of the atomized substance. The study is based on the previous work of the authors and the latest discoveries in this field.

## 2. Pulse Atomization

The higher the dispersion of the atomized decontaminating aerosol particles, the larger the surface of the interaction between particles and hazardous agents for the same mass of the atomized substance. Thus, rapid decontamination missions require not only to atomize an aerosol cloud as quickly as possible but also to obtain a minimum particle size.

**Figure 1:**
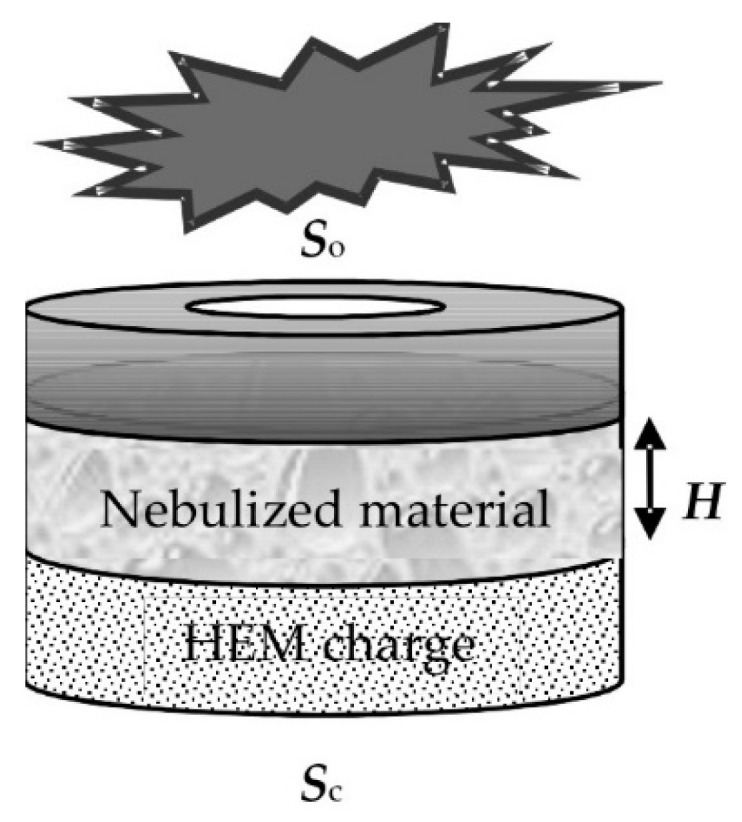
Scheme of the pulse atomizer design: an explosive charge is located in the lower part of the cylindrical body; a layer of the nebulized material is limited by an explosion-destroying membrane. *H* is the height of the material layer, *S_o_* is the area of the nozzle and *S_c_* is the cross-sectional area of the cylinder.

The original design of a pulse atomizer using HEM energy is depicted in Figure 1. In the explosion chamber, the base of the structure represents an HEM charge. After the charge is activated, the reaction products displace the atomized substance from a nozzle in the upper part.

### 2.1. Mathematical Model of the Pulse Atomization of a Liquid

The aerodynamic method of liquid atomization implies the breaking of a jet into droplets due to friction in the air. However, the aerodynamic atomization has a theoretical limitation on the size of the resulting droplets, depending on the jet velocity [24]. In this regard, a cloud of the highly dispersed aerosol may be quickly produced by a pulse generator using HEM energy, as presented by the authors earlier [25,26,27]. It has been shown that in contrast with aerodynamic atomization methods, the reduction in the droplet size during the pulse generation is caused by the cavitation process.

Following the author’s work [28], let us consider a mathematical description of the liquid aerosol generation in a cavitation regime.

Once an explosive charge is triggered, a shock wave is created in the liquid layer, which degenerates into a series of acoustic resonant oscillations with a wavelength λ = 2*H* and a frequency *f* = 2*c*/*H*. The reason is that the upper part of the structure is not open but limited. The relative displacement amplitude in the wave is as follows:

(1) $$A=\frac{1}{\pi c}\sqrt{\frac{2QM_{H}}{M_{l}}}$$

where *Q* is the heat of the HEM explosion (the specific energy), *M_H_* is the HEM mass, and *M_l_* is the liquid mass.

The maximum pressure attained in the liquid is determined in the instantaneous detonation approximation as $p=\frac{\left(\gamma_{H}-1\right)}{\gamma_{H}}\frac{QM_{H}}{V_{H}}$, where *V_H_* is the volume of the explosion chamber and γ*_H_* is the adiabatic exponent of combustion products.

The velocity of the liquid flowing out of the atomizer structure into the atmosphere at pressure *p_atm_* with account for the compression ratio ε = *S_o_*/*S_c_*, where *S_o_* is the area of the nozzle and *S_c_* is the cross-sectional area of the cylinder, is determined as

(2) $$v_{e}=\sqrt{\frac{2(p-p_{atm})}{\rho (1-\varepsilon^{2})}}$$

Under certain conditions, a cavitation flow occurs in this structure. The conditions for the cavitation regime establishing are governed by the cavitation number

(3) $$X=\frac{2(p-p_{s})}{\rho v_{e}^{2}}\leq 1,$$

where *p_s_* is the saturated-vapor pressure

With account for (2), the expression for cavitation condition (3) is written as

(4) $$X=\frac{\left(p-p_{s})(1-\varepsilon^{2}\right)}{\left(p-p_{atm}\right)}\leq 1$$

and for the cavitation to occur, the condition for the critical pressure produced by the HEM energy is specified as

(5) $$p\geq p_{cr}=\frac{p_{atm}-p_{s}(1-\varepsilon^{2})}{\varepsilon^{2}}$$

### 2.2. Cavitation and Dispersion of Droplets

Let us assume that the cavitation regime is induced by the acoustic waves propagating in a liquid medium. In such a regime, cavitation bubbles of the same diameter *D_c_* are formed and are evenly distributed in the medium.

The size of the cavitation bubble, occurring in the acoustic wave compression phase, is proportional to the amplitude

*D_c_* = *k*·*A*·*H* (6)

where the index of cavitation k represents the ratio of the volume of cavitation bubbles *V_c_* to the volume of the liquid *V_l_*, i.e., *k = V_c_/V_l_*. According to [28], for the developed cavitation regime, the coefficient *k* is in the range of 0.2–0.3.

Under continuous outflow conditions, the only variation in the cavitation bubbles with decreasing pressure is growth without collapsing [28]. If the bubbles are distributed evenly, each of them is surrounded by a liquid layer. After entering the atmosphere, the bubbles expand to a certain maximum size, determined by surface tension, and then collapse. Let us estimate the thickness of the bubble liquid wall at the moment of collapse. As the processes under consideration proceed at high velocities, the expansion of the cavitation bubble may be assumed to be adiabatic

(7) $$\frac{D_{\max}}{D_{c}}={\left(\frac{p}{p_{\min}}\right)}^{\frac{1}{3\gamma }}$$

where γ is the adiabatic exponent of the liquid vapor, *p*_min_ is the minimum pressure in the bubble at the moment of collapse, and *D*_max_ is the maximum bubble diameter before collapse.

The minimum pressure in the bubble before it collapses may be obtained from the ratio of the internal energy of the vapor in the bubble and the surface energy

(8) $$p_{\min}V_{c}=\sigma \cdot S_{c}$$

where *V_c_* is the volume of the cavitation bubble, *S_c_* is the surface area of the bubble, and σ is the surface tension, hence

(9) $$p_{\min}=\frac{6\sigma }{D_{\max}}$$

With account for (7) and (9), a transcendental equation is obtained for *D*_max_

(10) $$D_{\max}=D_{c}{\left(\frac{pD_{\max}}{6\cdot \sigma }\right)}^{\frac{1}{6\gamma }}=k\cdot A\cdot H{\left(\frac{pD_{\max}}{6\cdot \sigma }\right)}^{\frac{1}{6\gamma }}$$

The bubble sizes before expansion and at the moment of collapse are *D_c_* and *D*_max_, respectively. The outer diameter of the bubble with a liquid layer (before expansion) is denoted by *D_l_*_0_, and at the moment of collapse, *D_l_*_max_. The thickness of the liquid layer at the moment of collapse is specified as *h* = (*D_l_*_max_ − *D*_max_)/2. Taking into account (7) and the condition of equality of the liquid mass carried by the bubble inside the structure and after flowing out of the nozzle, $D_{l0}^{3}-D_{c}^{3}=D_{l\max}^{3}-D_{\max}^{3}$, the following expression is obtained for *h*

(11) $$h=\frac{D_{c}}{2}\left[{\left(\frac{1-k}{k}+\frac{p}{p_{\min}}\right)}^{1/3\gamma }-{\left(\frac{p}{p_{\min}}\right)}^{1/3\gamma }\right]$$

An analysis of high-speed shooting [24] shows that the bubble collapse is followed by the occurrence of droplets that correspond in magnitude to the thickness of the bubble wall. Thus, it may be assumed that the minimum particle size is equal to the thickness of the bubble liquid wall at the moment of collapse *D_p_* = *h*. Considering (8), the minimum droplet diameter is obtained

(12) $$D_{p}=\frac{k\cdot A\cdot H}{2}\left[{\left(\frac{1-k}{k}+\frac{p}{p_{\min}}\right)}^{1/3\gamma }-{\left(\frac{p}{p_{\min}}\right)}^{1/3\gamma }\right]$$

With account for (9) and (10), expression (12) determines the dependence of the minimum particle diameter on the pressure in the liquid *p*, the displacement amplitude in the wave *A*, the liquid layer thickness *H*, and the surface tension *σ*. The displacement amplitude is calculated using Formula (1).

The described procedure of the formation of droplets during the highly cavitated liquid atomization is fundamentally different from the dynamic destruction of jets in nozzles and atomizers caused by the oncoming air flow. Here, the droplet size depends primarily on the jet velocity with respect to the air and on the properties of the liquid, i.e., viscosity and surface tension.

### 2.3. Pulse Atomization of Powders 

We propose a new extension of the mathematical model. When modeling the atomization of a powder in the first approximation, the above-proposed model of the liquid atomization may be applied with two-phase losses neglected. Thus, the outflow velocity is determined by expression (2). It is improper to consider the development of cavitation in a powder, namely, in a two-phase flow of the powder and gaseous products of the HEM reaction. However, here again, a series of acoustic waves probably occur after the first shock wave in this mixture. Both the shock wave and the series of acoustic waves of sufficient intensity led to the destruction of the particles and their agglomerates.

Considering the particle of diameter *D_p_* located at a sound wave front and analyzing the acting forces, the threshold destruction intensity is calculated as [29]

(13) $$I_{t}=2W_{m}{\left(\frac{\sigma_{stp}}{D_{p}\cdot f\cdot \rho_{m}}\right)}^{2}$$

where σ*_stp_* is the ultimate strength of the particle, ρ*_m_* is the density of the dispersion medium, and *W_m_* = ρ*_m_·c* is the wave resistance of the medium. Note that the ultimate strength of the particle agglomerates is several orders of magnitude lower than that of a monolithic particle. For example, aluminum oxide has a high strength of about 500 MPa, while the microparticle agglomerates may be disintegrated at σ*_stp_* ≈ 75 MPa and less. The powder in the initial state is most likely agglomerated. 

Here, the dispersion medium is a two-phase mixture of gases and powder particles. Let us consider the atomization of nanostructured powders, which in the initial state are the agglomerates of nanoparticles. This type of powder is used when creating an aerosol with a maximum surface area of interaction with hazardous substances. In accordance with [30], such powders are considered as a new type of continuous medium, since the particle sizes are comparable to the sizes of gas molecules, and the bulk density of the powders is orders of magnitude lower than that of a compact substance. Such a medium may be represented as a pseudo-liquid, or a “heavy” gas, while the behavior of the nanopowders may be compared with that of the typical media studied in the relevant branches of mechanics. The speed of sound in the nanopowders, which has been determined in [31], is 50–70 m/s.

From Equation (13), one can obtain the expression specifying the minimum particle diameter for the agglomerate to be disintegrated at a given impact intensity *I*

(14) $$D_{p}=\frac{\sigma_{stp}}{f\cdot \rho_{m}}\sqrt{\frac{2W_{m}}{I}}$$

Expressions (12) and (14) allow one to determine the minimum possible particle sizes during the shock-wave atomization of the liquids and powders. The following properties may change during the atomization process: the pressure in the structure (in the pulse generator, the pressure drops as the substance flows out), the cavitation index, the impact intensity, and the frequency. When the bubble collapses, the droplets may be several times larger than the thickness of the bubble liquid wall. Moreover, the diameter of the powder particles occurring after its destruction in an acoustic wave may exceed the minimum determined by expression (14).

### 2.4. Size and Specific Surface Area of Particles

The efficiency of the interaction between aerosol particles and hazardous agents is provided not so much by the size of the particles but by their specific surface area. Real particles are not the perfectly smooth spherical objects but have pores and cracks. Therefore, the surface of a particle is determined not only by its diameter but also by the volume of irregularities, pores, and cracks. The surface of the particles is exactly where the interaction with hazardous substances occurs during adsorption, catalysis, or chemical reaction. As the effective diameter of the particles decreases, their surface area increases. That aside, one should note that during the atomization process, the cracks, micro- and nanopores may be filled with particle disintegration products and gaseous by-products of the HEM reaction, which eventually reduce their surface area.

The results of an experimental study of the specific surface area and the fineness of the nanostructured aluminum hydroxide powder (pseudoboehmite) using different methods of aerosol generation are presented in [32]. Three atomizer designs have been considered: an injector, a shock-wave atomizer (Figure 1), and a powder fire extinguisher. It has been shown that when the powder is atomized, its specific surface area always decreases but to a lesser extent when the shock-wave method of atomization is used.

The nanostructured powder under study has many pores and cracks of nano- and submicron sizes. Therefore, its initial specific surface area measured using the BET method is 257.8 m^2^/g, while the initial average particle diameter, the so-called Sauter mean diameter, is 48.4 µm. For all designs, the particles have reduced in size during the atomization process: down to 20 µm for the injection method and down to 10–11 µm for the pulse atomization. The reason is the mechanical fragmentation of particles and agglomerates during the process. Thus, a significant increase in the specific surface area of the particles is expected during the pulse atomization.

However, the specific surface area of the particles measured after atomization has shown a decrease by 8% in the case of the shock-wave atomizer and by 28% in other cases. In [32], this fact is explained by the occlusion of pores with nanoparticles resulting from the particle disintegration during the atomization and from the adsorption of gaseous products of the HEM reaction. The mechanical disintegration and pore occlusion during the powder atomization are observed in each considered case, but for the shock-wave method, these effects are partly offset by a large increase in the particle dispersion.

It has been noted that when a pyrotechnic charge with lower gas generation is used in the pulse atomization method, a decrease in the specific surface of the particles is several times smaller, which is explained by fewer gases filling the pores.

## 3. Results and Discussion

### 3.1. Parametric Study of the Model

**Figure 2:**
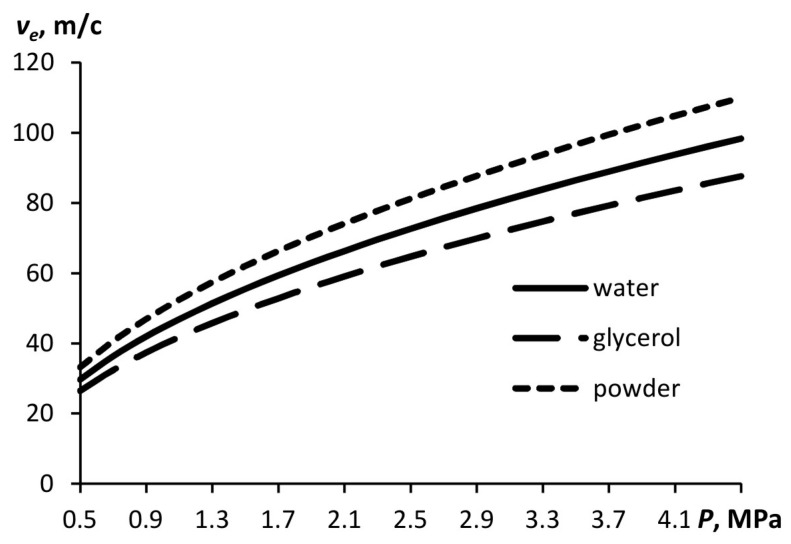
Outflow velocity as a function of pressure in the atomizer for substances with different densities (ε = 0.3).

Let us perform the calculations using the proposed model. Figure 2 shows the calculated flow velocity as a function of hydrostatic pressure for three atomized media with different densities. The substance with the highest density is glycerol, and with the lowest density, namely, with a bulk density of 800 kg/m^3^, is a powder. With increasing pressure and decreasing density, the flow velocity increases. The calculation is carried out for ε = 0.3.

**Figure 3:**
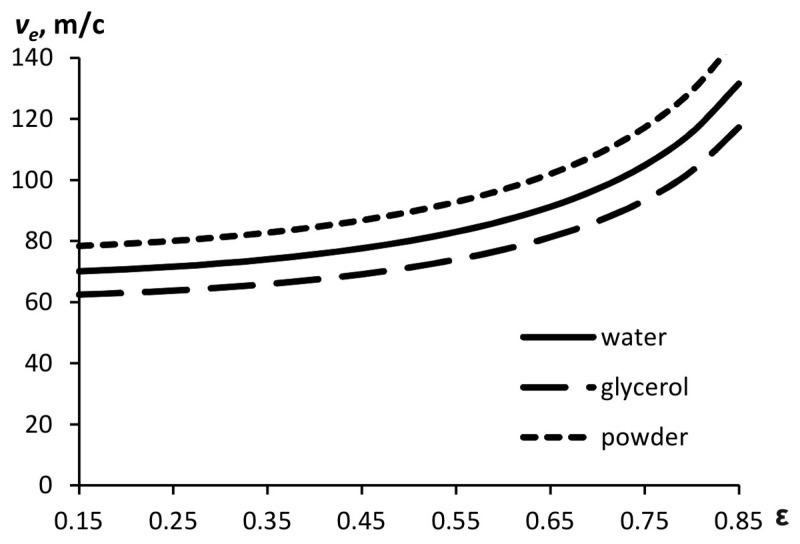
Outflow velocity as a function of compression ratio for substances with different densities (*P* = 2.5 MPa).

The dependence of the velocity on the compression ratio of the flow is shown in Figure 3. The calculation is made for *P* = 2.5 MPa.

**Figure 4:**
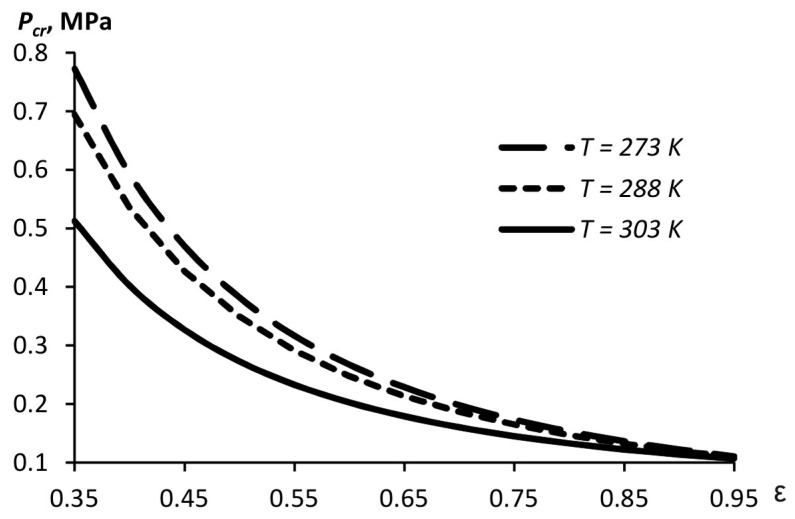
Critical cavitation pressure in the pulse atomizer design as a function of the compression ratio and temperature for aqueous solutions.

The critical pressure providing the cavitation in the liquid depends on the saturated vapor pressure and the compression ratio (5). When a narrow orifice is used, a higher pressure level is needed to create the cavitation due to the lower flow velocity as compared to that in the case of a wider orifice (Figure 4). The calculations use data for water since most disinfectant solutions are aqueous. The saturated vapor pressure changes with temperature.

Note that if the cavitation regime is not established, the size of the aerosol particles is determined by the jet destruction in the air, resulting in a lower dispersion of the aerosol. When designing the structures of pulse generators, one should strive to ensure the conditions for the liquid atomization with cavitation.

**Figure 5:**
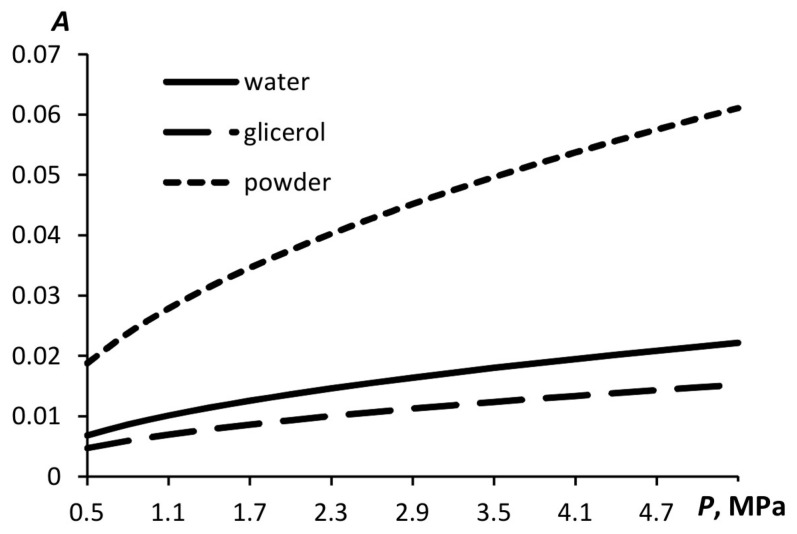
Relative displacement of the particles in a liquid as a function of pressure for water with a sound speed of *c* = 1481 m/s, glycerin with *c* = 1923 m/s, and powder with *c* = 60 m/s.

Figure 5 illustrates the dependence of the relative displacement in a liquid induced by the acoustic wave propagation for liquids with different densities and sound speeds, i.e., glycerin and water. The displacement of the powder particles during the acoustic wave propagation is also shown.

As shown above, in accordance with the proposed mathematical model, the smaller the particle displacement in the wave, the higher the droplet dispersion. Therefore, for the liquid with a higher speed of sound, the dispersion of droplets is expected to be higher under otherwise equal conditions. The dispersion of the solid aerosol does not depend on the displacement of the particles under acoustic action.

**Figure 6:**
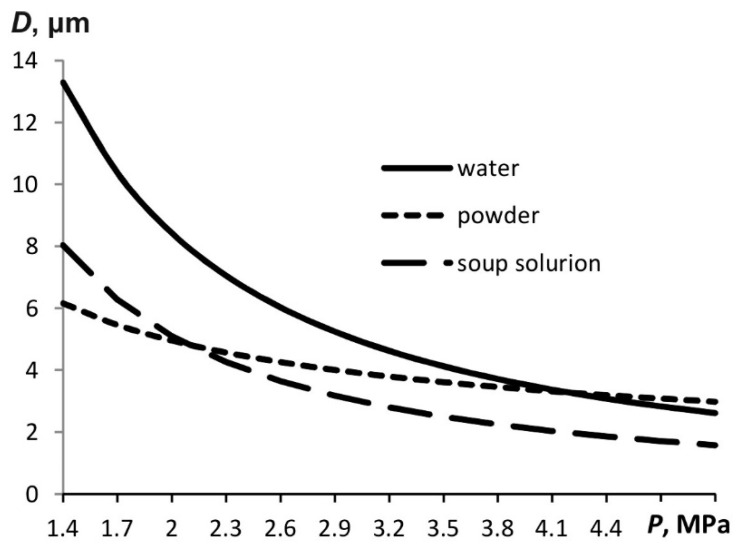
Minimal diameter of the aerosol particles during the pulse generation as a function of pressure.

The droplet size is affected not only by the particle displacement but also by the liquid surface tension if the proposed model is correct. The lower the surface tension, the more expanded the bubble carrying the liquid, which means that the particles will be smaller. Figure 6 shows the particle sizes of the aerosols of water, soap solution, and powder which have been calculated using the above-described model. The surface tension of the soap solution is 1.8 times less than that of the water. Therefore, the droplets formed are smaller than the water droplets. 

The dependence of the particle size on the pressure in the atomizer design for liquid aerosols is stronger than that for solid ones. Still, with increasing pressure, the particle size decreases in any case.

### 3.2. Comparison with Experiment

**Figure 7:**
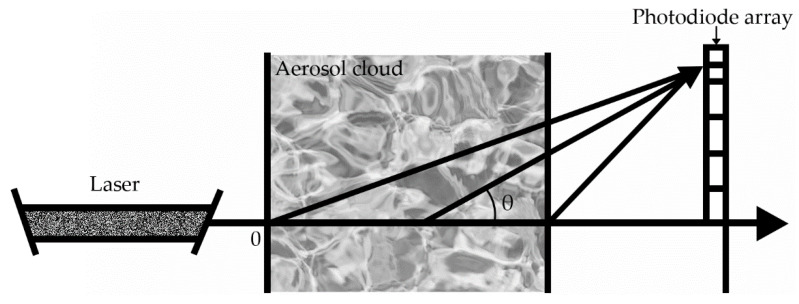
Scheme of interaction of laser radiation with an aerosol. The emitter is a helium-neon laser LG-78 with a wavelength of 0.63 μm. A photodiode array of seven FD-24K silicon photodiodes registers scattering in the range of angles θ = (0–15)°.

Liquid atomization is implemented experimentally using a pulse atomizer with the schematic design presented in Figure 1. To determine the concentration and size distribution function of the particles, a remote method is used based on the modified method of small angle scattering of probe radiation [33] (Figure 7). This method allows one to obtain the concentration and size distribution of the aerosol particles with a high time resolution.

This method is used on the assumption that the particles being considered are spherical, and multiple scattering is absent because of the small concentration of the particles. In the present work, we used a modified method of small-angle scattering based on the determination of the particle-size distribution function by solving a series of direct problems of aerosol optics. The essence of this method is that the sizes of aerosol particles are determined based on the measured small angle scattering indicatrix by exhaustion of the corresponding parameters of the distribution function.

**Figure 8:**
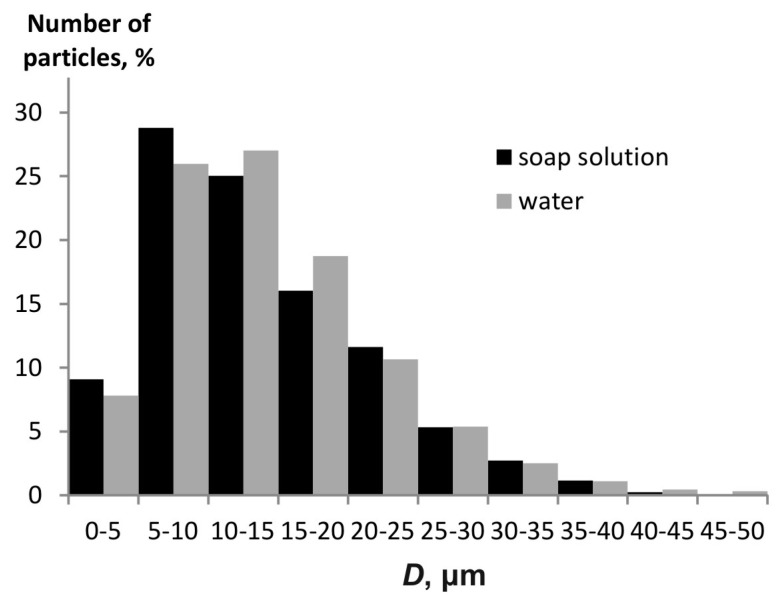
Histogram of the particle size distribution for the water aerosol and soap solution (*P* = 4.5 MPa).

Figure 8 shows histograms of the particle size distribution for the water aerosol and soap solution. Atomization is performed at a pressure of 4.5 MPa. The Sauter diameter is 22.1 µm for the water aerosol and 20.97 µm for the soap solution. In both cases, the finely dispersed aerosols with similar particle sizes are obtained. However, the soap solution particles are slightly smaller, which is compliant with mathematical modeling results (see, for example, Figure 6). At the same time, a noticeable fraction of particles has sizes in the range up to 5 μm, which corresponds to the calculation (it is the minimum particle size that is estimated in the calculations).

**Figure 9:**
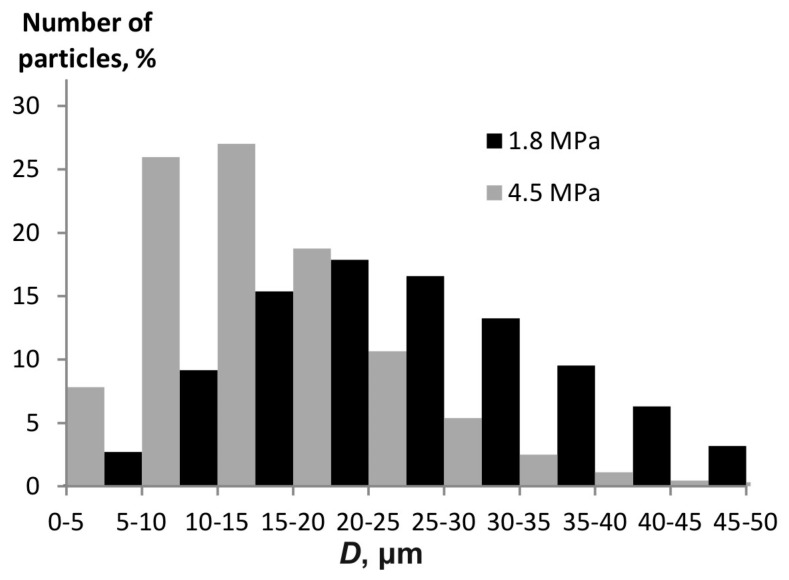
Histogram of the particle size distribution for a water aerosol at varying pressures in the pulse atomizer design.

Figure 9 presents a histogram of the water aerosol particle distribution at different pressures.

The Sauter diameter is 22.1 µm at a pressure of 4.5 MPa, while it is 38.4 µm at 1.8 MPa. This variation is consistent with the trend shown in Figure 5.

For a liquid with a higher surface tension, the particle size is smaller than for a liquid with a lower surface tension. This is also confirmed by the experiment with spraying water and soap solution.

The mathematical model makes it possible to precisely predict the minimum values of the particle diameter. With this in mind, it can be argued that the results of the calculations and experiment are shown to be in satisfactory agreement.

Thus, the presence of acoustic oscillations in the liquid atomized within the considered generator design has been shown to contribute to the development of cavitation. When flowing out into the air, the cavitated liquid breaks up into smaller droplets as compared to those in the case of a continuous liquid; this means that the aerosol has a high particle surface area.

The dispersion of the atomization is attributed to the cavitation during the droplet formation. The well-known procedure of dynamic destruction of jets in the air [24] gives estimates of droplet sizes of tens and hundreds of micrometers.

The pulse atomization method also affects the dispersion of the powder. Earlier experimental works of the authors have proved that the powder particle destruction into smaller particles, indeed, occurs during the pulse atomization, and, thus, the surface area of the particles increases. Still, one should note that in the atomization process, the pores and cracks of the particles are partly filled with the products of the HEM reaction and particle destruction.

## 4. Conclusions

The development of new methods to counteract environmental threats is an urgent problem of our time, which stimulated this work.

1. The pulse generation of aerosols with the use of HEM energy has been theoretically considered. It is assumed to be used when creating clouds of decontaminating aerosols interacting with hazardous substances in the air and on surfaces. In this regard, the particles of the generated aerosol should have a developed surface, i.e., a large specific surface area.
2. We have obtained expressions determining the critical pressure required for the cavitation development in a model design and the expression to assess the minimum droplet size depending on the atomization conditions and physical and chemical properties of the liquid.
3. We made calculations of the outflow velocity depending on the pressure in the atomizer and the compression ratio (geometrical characteristics of the atomizer). Velocity increases with pressure and increase in compression ratio non-linearly.
4. The calculation of the minimum particle diameter as a function of pressure shows that both for a liquid and for a powder, the diameter decreases non-linearly with increasing pressure. We conducted an experiment on liquid spraying at two values of pressure in the atomizer, which confirmed this trend.
5. We have proposed the procedure to reduce the size of particles during the pulse atomization, accounting for the effect of acoustic oscillations, and gave the expressions for assessing the minimum particle sizes.
